# NMR study of human macroPARPs domains: ^1^H, ^13^C and ^15^N backbone and side-chain chemical shift assignments of hPARP9 macro domain 1 (MD1) in the apo and in the ADPr bound states

**DOI:** 10.1007/s12104-026-10270-9

**Published:** 2026-06-12

**Authors:** Danai Moschidi, Nikolaos K. Fourkiotis, Sofia-Antigoni Tsatsouli, Aikaterini C. Tsika, Georgios A. Spyroulias

**Affiliations:** Department of Pharmacy, University of Patras, 26504 Patras, Greece

**Keywords:** hPARP9, Macro domain 1 (MD1), ADP-ribosylation, NMR spectroscopy

## Abstract

ADP-ribosylation is a reversible post-translational modification that regulates diverse cellular processes, including DNA damage repair, transcription, cell proliferation and innate immune responses, and is primarily catalyzed by members of the PARP family. While all 17 human PARPs contain a conserved C-terminal ADP-ribosyltransferase (ART) domain, only catalytically active members transfer ADP-ribose (ADPr) from nicotinamide adenine dinucleotide (NAD⁺) onto proteins or nucleic acids, and their N-terminal accessory domains, such as macro domains (MDs), WWE domains or RNA-binding motifs, mediate interactions that diversify PARP functions. Human PARP9 (hPARP9), known also as BAL1, is catalytically inactive due to sequence variations in catalytically important residues in the ART domain, but plays crucial roles in antiviral and antibacterial defense, stress responses and tumor progression through its heterodimeric interaction with the E3 ubiquitin ligase DTX3L. hPARP9 contains two tandem MDs (MD1 and MD2), with MD1 acting as a MacroD-type hydrolase “eraser” of mono-ADP-ribosylation (MARylation), while MD2 functions as an ADPr “reader”. Their different role in the ADP-ribosylation pathway highlights the importance of structural and functional characterization for understanding ADPr-mediated cellular signaling. In this study, we report the NMR backbone and side-chain resonance assignments of hPARP9 MD1 in both apo and ADPr bound states. In addition, the secondary structure predictions using TALOS+ server and the Chemical Shift Perturbation (CSP) analysis upon ADPr binding are presented. The latter illustrates the MD substrate’s accommodation mode and identifies the residues involved in ADPr binding, thus related to MDs’ hydrolytic activity.

## Biological context

ADP-ribosylation is a reversible post-translational modification (PTM) that regulates numerous cellular processes, including DNA damage repair, transcription, cell proliferation and innate immune responses (Palazzo et al. 2017; Lüscher et al. 2018). The modification is primarily catalyzed by members of the PARP family, which can generate either mono-ADP-ribosylation (MARylation) or poly-ADP-ribosylation (PARylation), depending on the enzymatic mechanism and the biological context (Luo and Kraus 2012; Lüscher et al. 2022; Wu et al. 2024). Although all 17 human PARPs share a conserved C-terminal ART domain, only the catalytically active members transfer ADPr units from nicotinamide adenine dinucleotide (NAD^+^) onto proteins or nucleic acids, thereby generating ADP-ribosylation-dependent signaling. Their diverse N-terminal accessory domains, such as macro domains (MDs), WWE domains or RNA-binding motifs, mediate interactions with nucleic acids, protein partners and other post-translational modifications (PTMs) (Suskiewicz et al. 2023). The modular architectures diversify PARP functions, enabling them to act as key regulators of genome stability, cellular stress signaling and antiviral defense (Lei et al. 2025).

The human PARP9 (hPARP9), also known as BAL1 (B-aggressive lymphoma 1), is catalytically inactive due to substitutions in the conserved H-Y-[E/I/L/Y] motif of ART domain, bearing a Q-Y-T triad instead (Vyas et al. 2014). Despite its lack of ART activity, hPARP9 is strongly implicated in immunity and stress responses through its heterodimeric interaction with the E3 ubiquitin ligase DTX3L, forming an interferon-inducible complex involved in viral RNA sensing, ubiquitination of viral proteins, STAT1 signaling activation and reinforcing other IFN-stimulated genes (Zhang et al. 2015; Xing et al. 2021). hPARP9 also contributes to antibacterial defense (Zhu et al. 2024) and is associated with tumor aggressiveness in lymphoma, breast cancer and glioma (Yan et al. 2013; Xu et al. 2020).

**Fig. 1:**
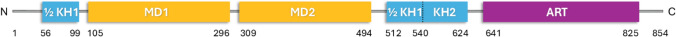
Domain organization of hPARP9 protein based on AlphaFold2 structural prediction (Suskiewicz et al. 2023). For the prediction, the protein sequence corresponding to the Uniprot accession number: Q8IXQ6 was used. KH indicates the KH domain, MD the macro domain and ART the ADP-ribosyltransferase domain.

The hPARP9 is a multi-domain protein that, in addition to its C-terminal ART domain, contains two tandem MDs, MD1 and MD2, and two KH domains, KH1 and KH2. According to AlphaFold2 structural prediction, the KH domains are separated and allocated in different segments in the primary sequence, but assemble into a compact conformation, although their exact boundaries and functions have yet to be fully delineated (Fig. 1) (Suskiewicz et al. 2023). Due to the presence of the macro domains, hPARP9 belongs to the macroPARP subfamily, which also includes hPARP14 and hPARP15, spanning their sequences tandems of three and two MDs, respectively, with each domain exhibiting distinct regulatory roles (Rack and Perina 2016; Fourkiotis et al. 2022; Suskiewicz et al. 2023). Specifically, macro domains are evolutionarily highly conserved domains adopting an α/β/α sandwich-like fold and play pivotal role in regulating ADP-ribosylation related pathways. They function either as “readers”, recognizing and binding free ADPr or ADP-ribosylated substrates, such as proteins and nucleic acids, or as “erasers”, binding and removing through hydrolytic cleavage of the ADPr from modified substrates to reverse the modification (Rack and Perina 2016).

Within hPARP9, the first macro domain (MD1) is considered a MacroD-type hydrolase that functions as an “eraser” of MARylation. hPARP9 MD1 hydrolyzes ADPr from MARylated protein substrates, including the ART domain of hPARP10, and from ADP-ribosylated RNA (Weixler et al. 2025). This activity distinguishes hPARP9 MD1 from the second domain, MD2, which binds ADPr, but does not display hydrolase activity, acting instead as a “reader” (Weixler et al. 2025). Given the central roles of hPARP9 in interferon signaling, ubiquitin-mediated antiviral defense and early DNA damage responses, the characterization of the conformation and the dynamics along with the biochemical properties of hPARP9 MD1 is essential for understanding how hPARP9 is implicated in ADP-ribosylation related processes (Yan et al. 2013; Zhang et al. 2015; Xing et al. 2021).

For this purpose, we report here the solution NMR study of hPARP9 MD1 domain in both free and ADPr bound forms to obtain the backbone and side-chain resonance assignments prior to its complete NMR conformational dynamics analysis. In addition to the crystal structures recently deposited by our research group in hPARP9 MD1 apo (PDB ID: 9QYD) and ADPr bound (PDB ID: 9QYE) states, NMR analysis provides valuable insights into the protein’s binding properties, to set the basis for an atomic-level investigation on how ADPr binding modulates structural flexibility and conformational behavior.

## Methods and experiments

### Construct design

The DNA sequence coding for the hPARP9 MD1 (residues 102–300) (Uniprot accession number: Q8IXQ6) was obtained, with codon optimization for *E. coli* expression, from GenScript (Piscataway, NJ). The codon optimized gene was amplified using PCR and then cloned into pETM-41 expression vector between the *Nco*I and *Not*I restriction sites. The coding sequence has been designed to produce the hPARP9 MD1 with a His_6_-tag followed by a MBP-tag and a Tobacco Etch Virus (TEV) cleavage site in the N-terminus. DNA sequencing was used to verify the obtained construct.

### Protein expression and uniform ^15^N and ^15^N/^13^C labeling

The pETM-41 plasmid encoding hPARP9 MD1 was transformed into *E. coli* EXPRESS BL21 (DE3) (Lucigen) cells. Few colonies were used to inoculate a *Luria–Bertani* (LB) pre-culture incubated overnight at 37 ˚C with shaking at 180 rpm. Then, 3 mL of the pre-culture were added in 0.5 L of M9 minimal medium (48 mM Na_2_HPO_4_, 22 mM KH_2_PO_4_, 8 mM NaCl) containing 0.5 g ^15^NH_4_Cl and 2 g unlabeled or ^13^C-D-glucose, 1 mL from a stock solution containing 0.5 mg/mL biotin and 0.5 mg/mL thiamine, 0.5 mL of 1 M Mg_2_SO_4_, 0.15 mL of 1 M CaCl_2_, 1 mL of trace elements solution (40 mM HCl, 50 mg/L FeCl_2_·4H_2_O, 184 mg/L CaCl_2_·2H_2_O, 64 mg/L H_3_BO_3_, 18 mg/L CoCl_2_·6H_2_O, 4 mg/L CuCl_2_·2H_2_O, 340 mg/L ZnCl_2_, 710 mg/L Na_2_MoO_4_·2H_2_O, 40 mg/L MnCl_2_·4H_2_O) and 50 μg/mL kanamycin. The culture was incubated at 37 ˚C with shaking at 180 rpm until the OD_600_ reached 0.6–0.8 values when 0.15 mM IPTG was added and incubated overnight at 18 ˚C.

### Protein purification and sample preparation

After 12 h of induction, the cells were harvested by centrifugation at 7,000 rpm for 10 min at 4 ˚C (Thermo Scientific^®^, Sorvall Lynx 6000). The cell pellet was resuspended using 25 mL lysis buffer (10 mM Imidazole, 50 mM Tris pH 7, 1 M NaCl), 10% glycerol, 0.1% Triton-X, 2 mM DTT and 10 μL of protease inhibitor cocktail (Sigma Aldrich^®^ P8849) as well 10 μL of DNase (10 mg/mL) were added and incubated for 10 min on ice. Then, the cells were sonicated (PMisonix^®^, Sonicator 4000) and centrifuged at 13,000 rpm for 45 min at 4 ˚C. The soluble fraction containing the His_6_-MBP-tagged hPARP9 MD1 was filtrated with 0.22 μm membrane filter and loaded onto a 5 mL Histrap™ FF affinity column (Cytiva) that had been previously equilibrated with 0.1 M NiSO_4_·7H_2_O and 5 column volumes (CV) binding buffer (10 mM Imidazole, 50 mM Tris pH 8, 500 mM NaCl). The elution of the protein was achieved using a step gradient with increasing concentration of imidazole (10, 20, 40, 100, 200, 400 mM in buffer also containing 50 mM Tris pH 8, 500 mM NaCl). The fractions containing the hPARP9 MD1 protein were concentrated using an Amicon^®^ Ultra 15 mL Centrifugal Filter membrane (nominal molecular weight cutoff 10 kDa), to final volume of 5 mL, and as well buffer exchange was performed from the imidazole-containing buffer to the TEV cleavage buffer containing 50 mM Tris pH 8, 300 mM NaCl, 2 mM DTT. After overnight incubation with 350 μL of TEV protease (1 mg/mL) at 4 °C, the sample was loaded again onto the 5 mL Histrap™ FF affinity column to remove both the cleaved His_6_-MBP-tag and the TEV protease. Then, the concentration of the cleaved protein fractions to final volume of 10 mL as well buffer exchange to the buffer containing 50 mM Tris pH 7, 20 mM NaCl, 2 mM DTT were performed. To remove any impurities, an anion exchange (Hitrap Q FF, Cytiva) was performed. The 10 mL protein sample was loaded onto the 5 mL Hitrap Q FF column and eluted using step gradient with increasing concentration of NaCl (20, 50, 100, 250, 500, 1000 mM in buffer containing 50 mM Tris pH 7, 2 mM DTT) in the flow-through and the 20 mM NaCl fractions. The eluted fractions were analyzed using SDS-PAGE (15%) and Coomassie staining. The fractions containing the purified protein were concentrated and buffer exchanged to the NMR buffer (10 mM HEPES pH 7.1, 50 mM NaCl, 2 mM TCEP, 2 mM EDTA) to final volume of 500 μL. In this NMR sample, 1 μL of proteases inhibitor cocktail (Sigma Aldrich® P8849), 10% D_2_O and 0.25 mM DSS (4,4-dimethyl-4-silapentane-1-sulfonic acid, Sigma Aldrich^®^) were added to the final volume of 552 μL. DSS is used as internal ^1^H chemical shift standard. Both apo and ADPr bound (molar ratio of hPARP9 MD1:ADPr – 1:5, Sigma Aldrich^®^) states were studied. The concentration of the ^15^N samples were 0.35 mM for both forms while the concentration of the double labeled ^15^N, ^13^C samples was 0.35 mM for the apo and 0.3 mM for the ADPr bound state.

### NMR data acquisition and processing

The labeled samples were placed into a 5 mm tube to record all NMR experiments at 298 K on a Bruker Avance III High-Definition four-channel 700 MHz NMR Spectrometer equipped with a cryogenically cooled 5 mm ^1^H/^13^C/^15^N/D Z-gradient probe (TCI). In Table 1, an overview of the NMR experiments and corresponding main parameters acquired for backbone and side-chain assignments for both free and ADPr bound form is provided. Specifically, the heteronuclear experiments 2D ^1^H,^15^N HSQC and 2D ^1^H,^13^C HSQC, 3D HN(CO)CA, 3D HNCA, 3D HN(CO)CACB, 3D HNCACB, 3D HN(CA)CO, 3D HNCO, 3D HNHA, 3D HBHA(CBCACO)NH, 3D aliphatic (H)CCH-TOCSY and 3D ^1^H,^15^N NOESY, 3D ^1^H,^13^C aliphatic NOESY and 3D ^1^H,^13^C aromatic NOESY were recorded and analyzed for the assignments of hPARP9 MD1 in both states. TopSpin 3.7.0 (Bruker Biospin) was used for the processing of the NMR data as well CARA 1.9.1.7 (Keller 2004) for the further analysis.

**Table 1:** Overview of NMR experiments and corresponding main parameters acquired at 700 MHz at 298 K for both hPARP9 MD1 states.

*hPARP9 MD1 apo & ADPr bound*	Time domain data size (points)			Spectral width/Carrier frequency (ppm)			NS
	t1	t2	t3	F1	F2	F3	
^1^H,^15^N HSQC	256	2048		44/120 (^15^N)	16/4.7 (^1^H)		4
^1^H,^13^C HSQC	512	2048		160/80 (^13^C)	14/4.7 (^1^H)		32
trosy HNCACB	96	40	1024	72/39 (^13^C)	44/117 (^15^N)	14/4.7 (^1^H)	16
trosy HN(CO)CACB	96	40	1024	72/39 (^13^C)	44/117 (^15^N)	14/4.7 (^1^H)	16
HNCA	80	40	1024	42/55 (^13^C)	44/117 (^15^N)	14/4.7 (^1^H)	8
HN(CO)CA	80	40	1024	42/55 (^13^C)	44/117 (^15^N)	14/4.7 (^1^H)	8
HNCO	64	40	1024	18/175 (^13^C)	44/117 (^15^N)	14/4.7 (^1^H)	8
HN(CA)CO	64	40	1024	18/175 (^13^C)	44/117 (^15^N)	14/4.7 (^1^H)	8
HNHA	48	96	1024	35/117 (^15^N)	14/4.7 (^1^H)	14/4.7 (^1^H)	16
HBHA(CBCACO)NH	112	40	1024	8/4.7 (^1^H)	44/117 (^15^N)	14/4.7 (^1^H)	16
(H)CCH-TOCSY	128	48	1024	80/39 (^13^C)	80/39 (^13^C)	14/4.7 (^1^H)	16
^1^H, ^15^N NOESY	232	48	2048	14/4.7 (^1^H)	40/117 (^15^N)	14/4.7 (^1^H)	16
^1^H, ^13^C NOESY aliphatic	192	64	1024	14/4.7 (^1^H)	80/39 (^13^C)	14/4.7 (^1^H)	8
^1^H, ^13^C NOESY aromatic	144	32	2048	14/4.7 (^1^H)	39/127 (^13^C)	14/4.7 (^1^H)	8

### Extent of assignments and data deposition

**Fig. 2:**
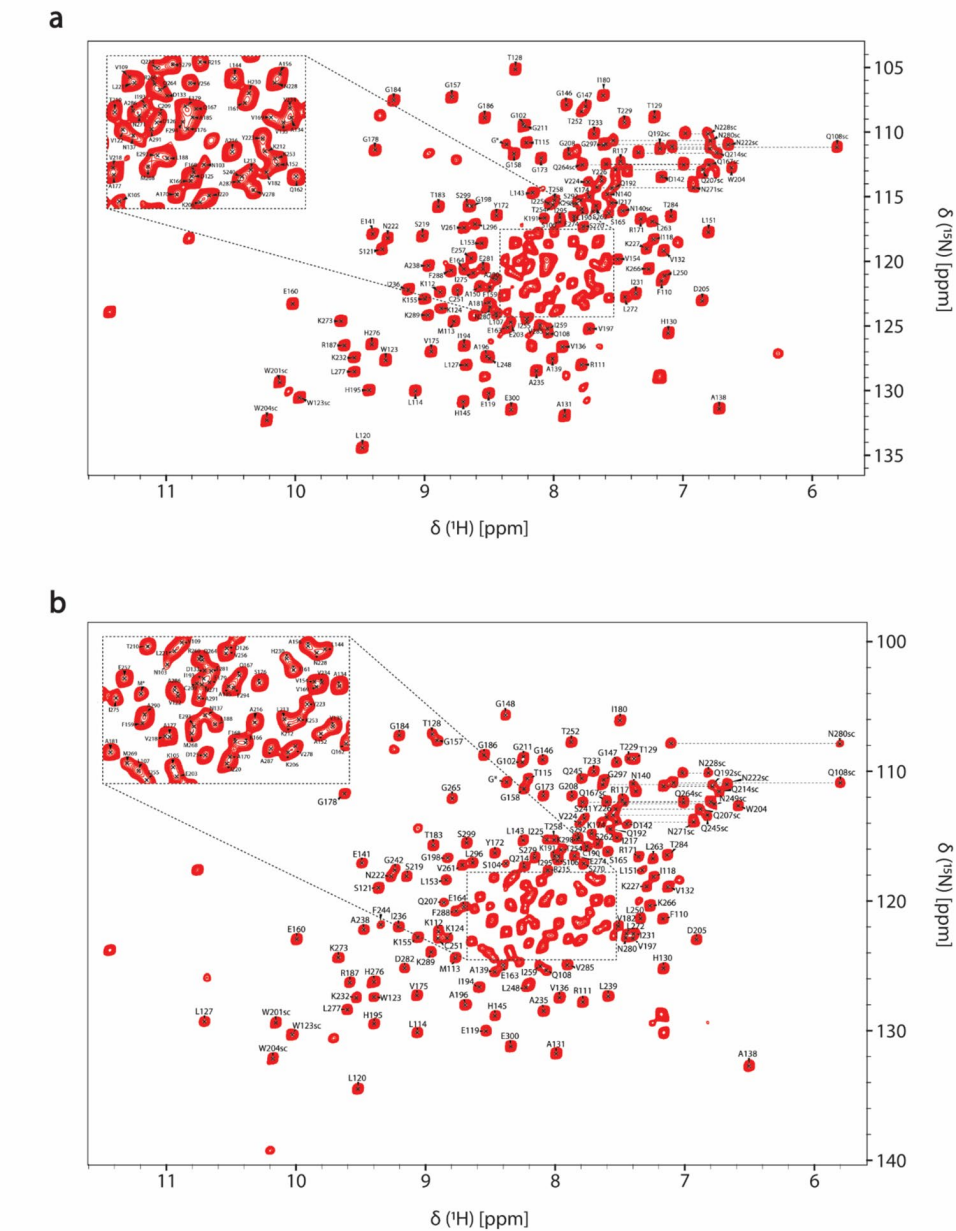
Annotated 2D ^1^H,^15^N HSQC spectra of hPARP9 MD1 in (**a**) apo state and (**b**) ADPr bound (molar ratio hPARP9 MD1:ADPr – 1:5) states recorded at 298 K with a 700 MHz NMR Spectrometer. The reported amino acids are numbered according to the native sequence, residues 102–300, while the cloning and cleavage artifact residues are labeled with asterisks. The side-chains of Asn, Gln and Trp resonances are labeled with “sc”. The unassigned resonances in both spectra correspond to residues that could not be assigned due to insufficient triple resonance data.

The studied polypeptide contains four cloning and TEV cleavage site artifact amino acids (GAMG) in the N-terminal region which were not considered in sequence numbering. The 2D ^1^H,^15^N HSQC spectra for both forms are shown in Fig. 2. Both are well-dispersed concluding that hPARP9 MD1 protein is well-folded in both states. The obtained assignments of the hPARP9 MD1 apo of the native sequence (102–300 a.a) (Fig. 2a) reached 91% of ^1^H^N^/^15^N backbone pairs, 95% of ^13^CO, 95% of ^13^Cα and 96% of ^13^Cβ chemical shifts and 75.2% of the total atoms of side chains. For the ADPr bound state (Fig. 2b), the corresponding percentages for the backbone and side-chain assignments were elevated to 95% of the ^1^H^N^/^15^N backbone pairs, 96% of ^13^CO, 97% of ^13^Cα and 96% of ^13^Cβ chemical shifts and 76.2% of the total atoms of side chains. The unassigned ^1^H^N^/^15^N resonances in the 2D ^1^H,^15^N HSQC spectra correspond to residues that could not be assigned due to insufficient spectral information obtained from the triple resonance experiments. In both states, except for the existing 7 prolines, these correspond to the residues L149, R200-M202, I243, F246 and N249 of the native sequence as well as the first two artifact residues GA. In hPARP9 MD1 apo state, the ^1^H^N^/^15^N peaks of the residues S104, G148, Q207, L239, S241, G242, F244, Q245, G265, M269 and D282 as well the artifact methionine residue were also missing from the 2D ^1^H,^15^N HSQC spectrum compared to the ADPr bound state in which they were assigned. Except for the three residues G148, Q207 and D282 located in the α-helices α1, α2 and α4, respectively, of the protein, as well the S104, which in the crystal structure (PDB ID: 9QYD) was not modeled, the remaining unassigned residues are located in two loops. Specifically, the missing residues G265 and M269 are located in the α3-β7 loop while the other 5 residues in the β6-α3 loop binding the phosphate of ADPr. On the other hand, in ADPr bound form, apart from the unassigned residues, only two ^1^H^N^/^15^N peaks of the residues A150 located in the α1 helix is broadened beyond detection and S240 located in the β6-α3 loop disappeared based on the titration data. The appearance of the residues in the specific region upon ADPr binding, which are missing in the free form, suggests that the structure of hPARP9 MD1 becomes more rigid in presence of ADPr, consistent with NMR observations for the macro domain of alphaviruses MAYV (Tsika et al. 2019), VEEV (Makrynitsa et al. 2019) and CHIKV (Tsika, Gallo, et al. 2022). This behavior contrasts with the reported data for the macro domains of the hPARP14 (Fourkiotis et al. 2022), the Rubella (Moschidi et al. 2025), the SARS-CoV-2 (Cantini et al. 2020), the SARS-CoV and the MERS-CoV (Tsika et al. 2022). Additionally, analysis of the ^13^Cβ and ^13^Cγ chemical shifts of proline residues of hPARP9 MD1 indicates that six out of seven prolines adopt *trans* conformation based on established reference values (Schubert et al. 2002). In contrast, P237, located in the β6-α3 loop, adopts a *cis* conformation with no evidence of *cis*–*trans* isomerization based on the NMR data of the neighboring residues.

**Fig. 3:**
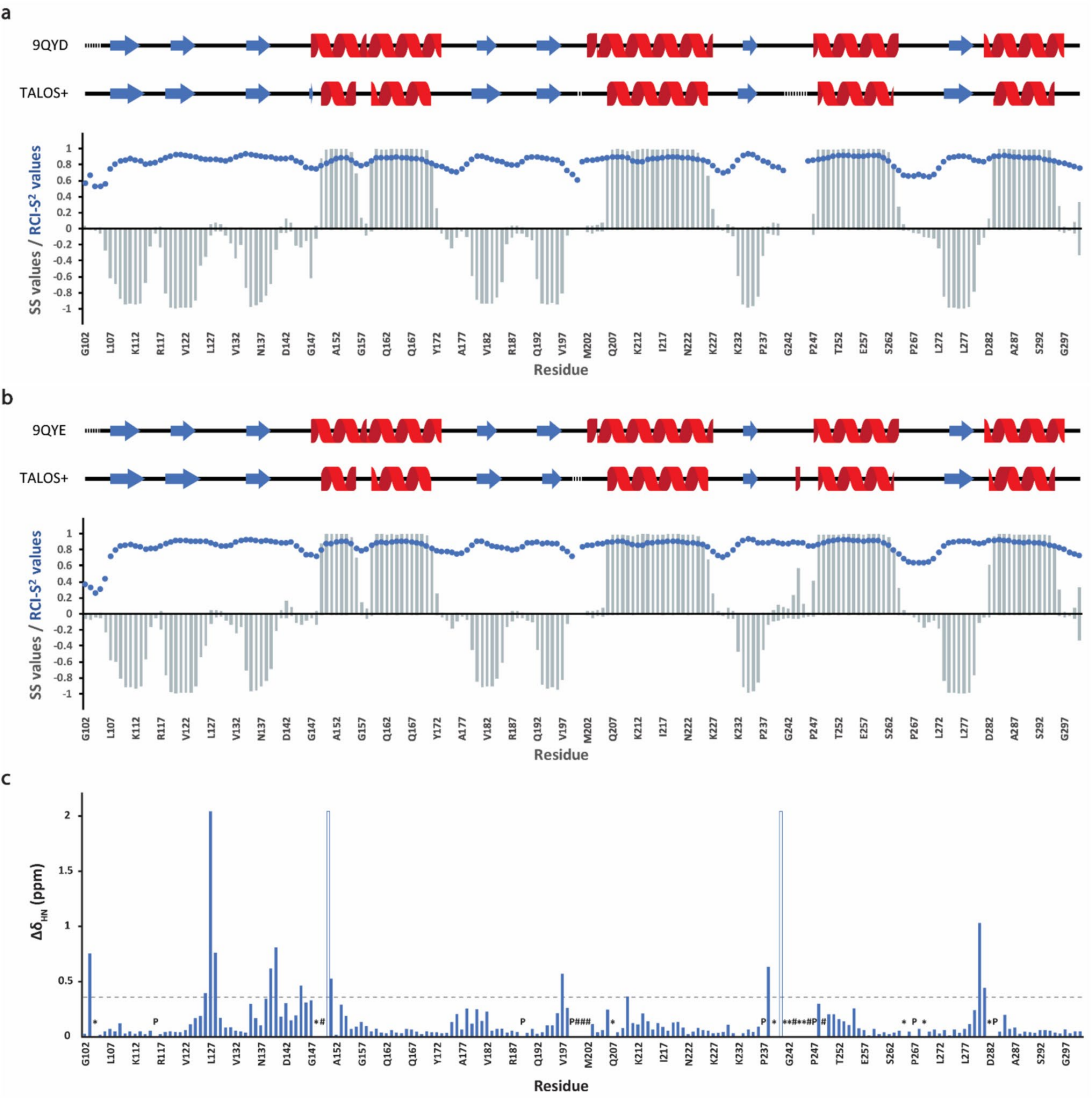
Secondary structure (SS) analysis and predicted S^2^ values of hPARP9 MD1 in (**a**) free form and (**b**) ADPr bound form and (**c**) Chemical Shift Perturbation (CSP) analysis of hPARP9 MD1 in the absence and presence of ADPr. The reported amino acids for hPARP9 MD1 are numbered in accordance with the native sequence. (**a**, **b**) Upper: Secondary structure elements derived from the corresponding crystal structures (apo state: 9QYD; ADPr bound state: 9QYE). Middle: Secondary structure predictions based on the experimental backbone NMR chemical shifts using the TALOS+ server (Shen et al. 2009). Red cartoon represents the α-helix, blue arrow the β-strand and dashes correspond to the unmodeled (upper) or unpredicted (middle) regions. Lower: Plot with the α-helix (positive) and β-strand (negative) SS values predicted using the TALOS+ server (Shen et al. 2009) for each residue. The SS values for β-strands propensities are displayed as negative for visualization purposes. Values close to 1 or -1 denote a strong propensity for α-helix and β-strand, respectively. The flexibility of hPARP9 MD1 is highlighted by the predicted order parameter (Random Coil Index (RCI) derived S^2^ (RCI-S^2^) values) shown with blue dots provided by TALOS+ server (Shen et al. 2009). (**c**) CSP values were calculated using the equation Δδ_ΗΝ_ = √[Δδ_H_^2^ + (Δδ_N_^2^)/25] and the threshold, shown as a horizontal dashed line, was defined as the mean plus a standard deviation. Empty bars indicate residues missing in ADPr bound state, (*) denotes residues missing in the apo state, (#) denotes residues missing in both states and P represents prolines.

Using the NMR backbone chemical shifts and TALOS+ server, the secondary structure prediction for both states of hPARP9 MD1 was performed (Shen et al. 2009). In Fig. 3 (a) and (b), these results are shown comparatively with the secondary structure elements of the crystal structures for both states of hPARP9 MD1, using the PDB ID: 9QYD for free form and PDB ID: 9QYE for ADPr bound form, concluding that they are in good agreement. In both crystal structures, two consecutive glycine residues (G157-G158) within the α1 helix introduce a pronounced bend in the helix. This region was the only one not correctly predicted by the TALOS+ server.

As the other human and viral macro domains, hPARP9 MD1, regardless of the state, is organized in an α/β/α sandwich-like fold with β/β/β/α/β/β/α/β/α/β/α topology. Moreover, TALOS+ server provides the Random Coil Index (RCI) derived S^2^ values (RCI-S^2^) (Fig. 3(a) and (b), blue dots), a parameter that is consistent with the flexibility of the protein. The RCI-S^2^ values of the rigid secondary elements α-helices and β-strands of the protein are close to 1, whereas the corresponding values of the loops and the N- and C- terminal regions are lower which shows a degree of flexibility.

Furthermore, Fig. 3(c) depicts the Chemical Shift Perturbation (CSP) analysis and the regions affected by the ADPr addition correspond to residues forming the moiety of ADPr binding. Looking closer, the residues of the β2-β3 loop, residues D125 to D133, known as adenine binding loop, and N280 located between β7 strand and α4 helix exhibit the higher CSP values among the sequence. In addition, the regions with notable CSP values correspond to the residues located in the catalytic loop β3-α1, responsible for the ADPr removal from substrates, N140 to G146, the first residues of α1 helix as well as the residues located in the β6-α3 loop that bind the phosphate groups of ADPr. This CSP analysis shows that regions within the ADPr-binding cavity are significantly perturbed upon ligand binding, suggesting conformational adjustments that accommodate the ligand and contribute to structural stabilization.

All the chemical shift values of hPARP9 MD1 apo and ADPr bound states were deposited in the Biological Magnetic Resonance Data Bank (BMRB) (https://bmrb.io) under accession numbers 53617 and 53618, respectively.

## Data Availability

All the chemical shift values of hPARP9 MD1 apo and ADPr bound states were deposited in the Biological Magnetic Resonance Data Bank (BMRB) under accession numbers 53617 and 53618, respectively.
